# Hip arthroplasty for patients with chronic renal failure on dialysis

**DOI:** 10.1038/s41598-023-30283-x

**Published:** 2023-02-27

**Authors:** Sang-Min Lee, Won Chul Shin, Seung Hun Woo, Tae Woo Kim, Do Hyung Kim, Kuen Tak Suh

**Affiliations:** 1 Department of Orthopaedic Surgery, Pusan National University Yangsan Hospital, 20 Geumo-ro, Mulgeum-eup, Yangsan, 626-770 Gyeongsangnam-do South Korea; 2 Research Institute for Convergence of Biomedical Science and Technology, Pusan National University School of Medicine, Yangsan, South Korea; 3 Department of Orthopaedic Surgery, Sehung Hospital, Busan, South Korea

**Keywords:** Pain, Bone, Kidney

## Abstract

An increasing number of chronic renal failure patients are experiencing hip joint disorders. This study aimed to analyze the outcomes of hip arthroplasty in chronic renal failure patients undergoing dialysis. Of 2364 hips that underwent hip arthroplasty during 2003–2017, 37 were retrospectively examined. Radiological and clinical outcomes of hip arthroplasty, and development of local and general complications during follow-up and their associations with dialysis duration were analyzed. The mean patient age, follow-up duration, and bone mineral density T-score were 60.6 years, 36.6 months, and − 2.62, respectively. Osteoporosis was noted in 20 cases. Most patients who underwent total hip arthroplasty with a cementless acetabular cup implant exhibited excellent radiological outcomes. There were no changes in femoral stem alignment, subsidence, osteolysis, and loosening. Thirty-three patients had an excellent or good Harris hip score. Complications developed in 18 patients within 1 year postoperatively. General complications developed in 12 patients at > 1 year postoperatively; no patient experienced local complications. In conclusion, hip arthroplasty for chronic renal failure patients on dialysis yielded excellent radiological and satisfactory clinical outcomes but may be associated with postoperative complications. Careful preoperative treatment planning and overall postoperative management are required to reduce the complication risk.

## Introduction

The incidence of chronic renal failure (CRF) has been steadily increasing; consequently, an increasing number of CRF patients are experiencing hip joint disorders^1,2^. Alem et al. estimated the incidence of femoral neck fractures among patients on dialysis to be 7 per 1000 person-years for men and 13 per 1000 person-years for women; these values were four times higher than those of the control groups^3^. In particular, bone quality in CRF patients on dialysis is often poorer than that of other patients. Also, CRF patients on dialysis show stronger associations with comorbidities (including diabetes mellitus and hypertension) and are often immunosuppressed. These patient characteristics can lead to poor surgical outcomes and poor prognoses after hip arthroplasty^3,4^. Despite the high risks associated with the surgical treatment of CRF patients on dialysis, debilitating joint conditions, including hip osteoarthritis, femoral head osteonecrosis, and traumatic hip fractures (e.g., femoral neck fracture), may necessitate surgical management and often constitute indications for hip arthroplasty^2,5–8^. Therefore, although hip replacement is more commonly performed for patients with chronic kidney disease (CKD), studies assessing the outcomes have reported variable success rates^2,5–11^.

Owing to the poor bone quality of CRF patients on dialysis, suitable stable fixation methods have not been identified. Studies closely investigating such methods had insufficient statistical power. Moreover, the available studies have not focused on the clinical analyses and complications after hip replacement. Therefore, we analyzed the radiological and clinical outcomes and complications of CRF patients on dialysis who were treated with total hip arthroplasty (THA) or bipolar hemiarthroplasty (BH) using a cementless acetabular cup with a proximally coated or cemented stem. The purpose of this study was to analyze the surgical outcomes and their association with dialysis duration.

## Methods

### Patients

Overall, 2364 hip joints were repaired with THA or BH at our hospital between January 2003 and December 2017. There were 187 CRF patients, including those undergoing hemodialysis or peritoneal dialysis; of these, the 153 patients who were not on dialysis were excluded. Finally, the data surrounding 37 arthroplasties in 34 patients (3 patients underwent bilateral treatment) were prospectively collected and retrospectively analyzed.

Data on associated comorbidities were collated from medical records, including the history recorded at the time of admission and preoperative evaluation. For all 37 cases, preoperative dual-energy X-ray absorptiometry was performed, and bone mineral density was measured to evaluate bone quality. Preoperative risk was evaluated according to the American Society of Anesthesiologists guidelines^12^.

### Ethics approval and consent to participate

This study followed the World Medical Association Declaration of Helsinki and strengthening the reporting of observational studies in epidemiology (STROBE) guidelines for cohort studies. All procedures performed in studies involving human participants were in accordance with ethical standards, patient information was reviewed by the university human subjects committee and written informed consent was obtained from all the patients. All experimental protocols were approved by our institutional committee (Pusan National University Yangsan Hospital, Approval No. 05-2020-024).

### Surgical technique and postoperative rehabilitation

Before surgery, all patients regularly underwent dialysis three times per week, and their serum creatinine and electrolyte levels were monitored in coordination with the nephrologist. After surgery, dialysis was continued at the same frequency. The antibiotic dose administered was determined based on kidney function, and the surgical drain was removed on postoperative day 2 for all patients.

A single senior surgeon performed all the procedures. Patients aged < 75 years with preserved cognition and ambulation underwent THA, whereas those aged > 75 years with poor cognition and ambulation underwent BH. A preoperative on-screen template^13^ was used and reviewed in all cases to accurately determine the size of the prosthetic implant and to correct any discrepancies in the patients’ leg length measurements. A posterolateral approach was used, and posterior soft tissue repair was performed for all patients^14^.

During THA, either the hemispheric cementless Trilogy (Zimmer, Warsaw, IN, USA) or Continuum (Zimmer) acetabular cup was implanted. When necessary, acetabular screws were additionally used for fixation. The Trilogy cup has a highly cross-linked polyethylene liner with a metal or ceramic prosthetic head, whereas the liner and the head of the Continuum cup are made of ceramic. Furthermore, a tapered fiber metal (Zimmer) was used as the cementless femoral stem, or the VerSys Heritage stem (Zimmer; with a centralizer in the distal portion) was utilized as the cemented femoral stem. The cementless stem was used when the bone quality was good and the initial press fitting was possible^10,13^, and the cemented stem was used when the cementless stem fixation could not be achieved because of a wide femoral isthmus or poor bone quality.

During postoperative rehabilitation, quadriceps exercises were initiated on postoperative day 1 and partial weight-bearing ambulation using a crutch was started on postoperative day 2. Full weight-bearing ambulation was allowed within 3 months of surgery for all patients. In accordance with nephrological protocol, a nephrologist performed the dialysis in a dialysis clinic after the patients were discharged. To prevent venous thromboembolism, low-molecular-weight heparin (LMWH) was administered to all patients except those with known endogenous bleeding risks. LMWH was administered to patients with hip fractures preoperatively and up to 2 weeks postoperatively (i.e., until discharge); for patients who underwent elective surgery, including those with osteoarthritis or osteonecrosis^15–17^.

### Radiological assessment

The postoperative outcomes, including osteolysis and migration, subsidence, or loosening of the prosthesis, as well as stability of the acetabular cup and femoral stem, were analyzed during follow-up radiological assessments.

For post-THA patients, vertical migration of the acetabular cup was assessed by measuring the change in vertical distance between the center of rotation (COR) of the cup and both ends of the teardrop. Horizontal migration was evaluated by estimating the change in horizontal distance between the vertical lines passing from the COR of the cup and onward through the center of the teardrop. Differences of > 2 mm in the vertical and horizontal migration between the final follow-up images and the postoperative plain radiographs associated with a change of > 5° in the pelvic tilt indicated significant prosthetic loosening. Furthermore, prosthetic loosening was considered to have occurred when the postoperative imaging revealed significant vertical or horizontal migration, a change in the acetabular cup’s inclination, or continuous, progressively widening radiolucent lines measuring > 2 mm around the acetabular cup^18^. Acetabular erosion and proximal migration of the cup indicated loosening in patients who underwent BH.

During the preoperative assessment of the femoral stem, plain radiographs were used to determine the proximal femoral form according to the Dorr classification^19^. Postoperatively, stable fixation of the cementless prosthetic femoral stem was evaluated by examining the bone ingrowth, stability of the fibrous fixation, and degree of instability of the prosthesis at the final follow-up, as suggested by Engh et al.^20^. As described previously^21,22^, femoral stem subsidence was measured as the distance from the medial and proximal notches on the microporous material to the proximal surface of the lesser trochanter, and it was considered significant when the difference between the two was > 5 mm and when radiolucent lines were observed in > 50% of the implant surface. Osteolysis was defined as a localized cystic erosion of the dilated cortical bone with a diameter of > 5 mm^23^. Progressive subsidence or migration of the femoral stem or radiolucent lines surrounding the femoral stem extending to > 2 mm indicated radiological loosening of the femoral stem^20^. The Harris classification was used to evaluate the cemented femoral stem^24^, and definite loosening was defined by radiological signs, including femoral stem subsidence, fracture involving the cement or stem, and progression of the radiolucent lines at the interface between the cement and femoral stem. Probable loosening was defined as progressive radiolucent lines observed at the overall interface between the cement and femoral stem. Possible loosening was defined as radiolucent lines covering 50–100% of the interface between the cement and femoral stem.

### Clinical assessment

The Harris hip score was used to evaluate the clinical outcomes^25^. The clinical outcomes were assessed preoperatively and at the final follow-up. The preoperative Harris hip scores of patients with hip fractures were excluded, and the final follow-up score was assessed. The preoperative Harris hip scores could not be obtained in patients with hip fractures as pain would cause interference with the assessment, thus were excluded. The Harris hip score was obtained at final follow-up for each patient. Scores ranged from 0 to 100, with 90–100, 80–89, 70–79, and < 70 points indicating excellent, good, fair, and poor outcomes, respectively.

Clinical analysis was performed by reviewing the patients’ medical records regarding the length of hospital stay, operative time, blood loss volume, requirement for intraoperative transfusion, preoperative dialysis duration, and total dialysis duration.

### Assessment of complications

Complications were classified as either local or general and were further categorized as early (developing at < 1 year postoperatively) or late (developing at > 1 year postoperatively). Local complications included surgical site infections, periprosthetic fractures, and dislocations requiring additional postoperative treatment. General complications included death, obvious worsening of underlying comorbidities, acute kidney insufficiency (AKI) requiring additional treatment other than the regular dialysis, venous thromboembolism, cardiopulmonary or urinary complications, and multi-organ failure. Furthermore, we analyzed the association of the development of general and local complications with preoperative and total dialysis durations.

### Statistical analysis

Continuous variables are expressed as means and standard deviations (SD), whereas categorical variables are presented as frequencies and percentages. Differences in clinical values at various time points were analyzed using the Wilcoxon signed-rank test. All statistical analyses were performed using SPSS software version 18 (IBM Corp., Armonk, NY, USA). A *P* value of < 0.05 was considered statistically significant.

## Results

### Patients

The mean age of the patients was 60.6 years (SD, ± 13.5 years; range 20–81 years), and the total study group included 15 (44.1%) men and 19 (55.9%) women. The mean follow-up duration (except for patients who died early) was 36.6 months (SD, ± 27.2 months; range 12–107 months). Altogether, there were 31 patients undergoing hemodialysis and 3 patients undergoing peritoneal dialysis. The mean preoperative dialysis duration was 34.2 ± 42.0 months (range 1–180 months), and the mean total dialysis duration was 57.1 months (SD, ± 46.8 months; range 13–198 months). A cementless proximally coated femoral stem was used in all 25 (100%) cases of THA, while a cemented femoral stem was used in 5 out of 12 (41.7%) cases of BH. The mean T-score indicating bone mineral density was -2.62 (SD, ± 1.15; range − 4.1 to − 0.5). Osteoporosis (defined as a T-score of < -2.5) was observed in 20 (54.1%) cases. Furthermore, 31 out of 34 patients had concurrent comorbidities other than CRF (Table 1).

**Table 1:** Demographic data of the study patients.

	No. of cases
Type of surgery	
Total hip arthroplasty	25
Bipolar hemiarthroplasty	12
Diagnosis	
Femoral neck fracture	24
Femoral head osteonecrosis	8
Hip osteoarthritis	3
Hip rheumatoid arthritis	2
Bone mineral density (T-score)*	
Normal (> − 1.0)	8
Osteopenia (− 1.0 to -2.5)	9
Osteoporosis (≤ − 2.5)	20
Comorbidities	
Hypertension	28
Diabetes mellitus	21
Cerebrovascular disease	9
Coronary artery occlusive disease	8
Liver disease	2
Rheumatoid arthritis	2
Prostate cancer	1
ASA score	
I	0
II	15
III	21

*ASA* American Society of Anesthesiologists.

*Estimated using dual-energy X-ray absorptiometry.

### Radiological outcomes

**Figure 1:**
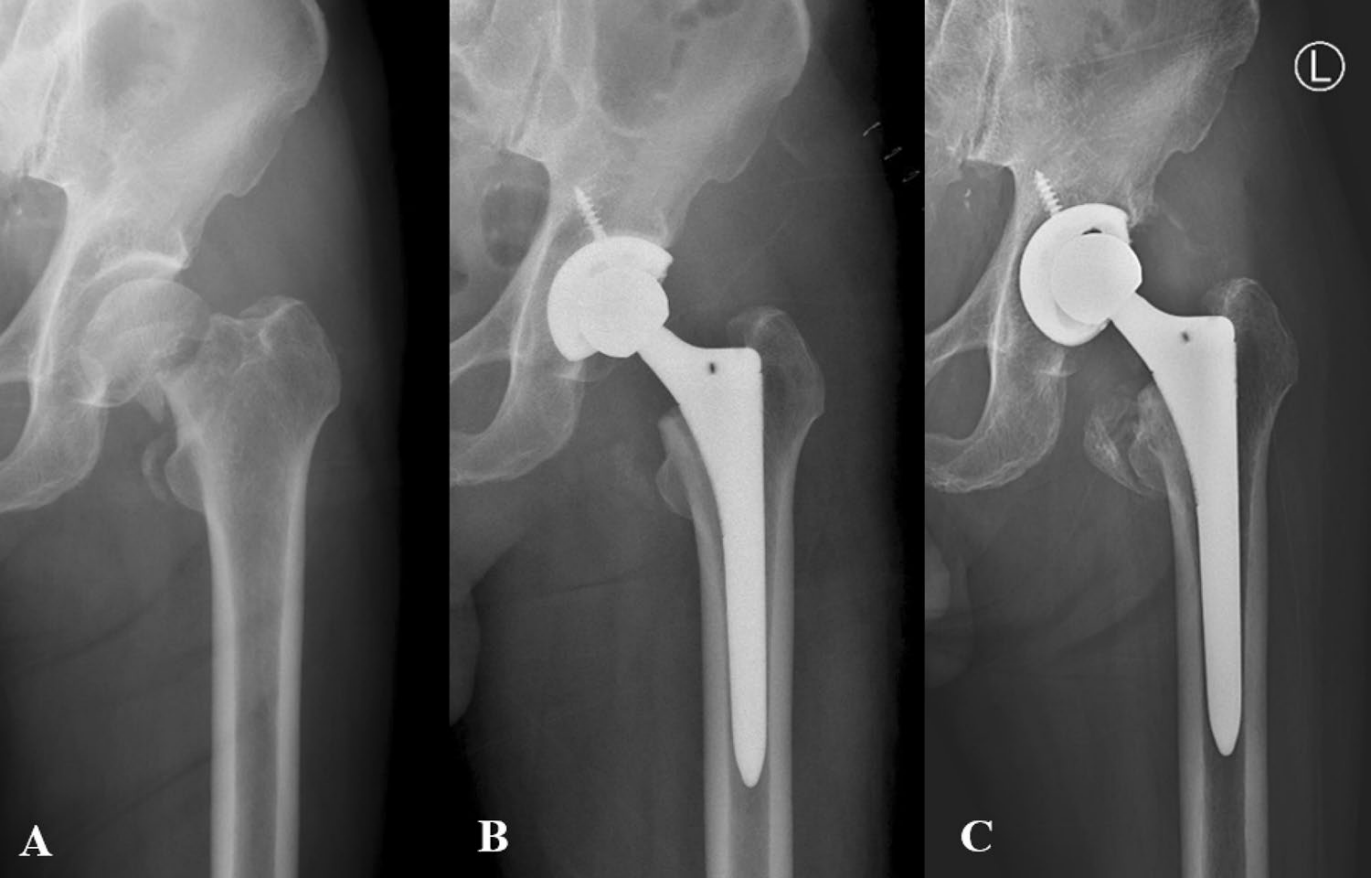
Serial radiographs of a 71-year-old patient who underwent total hip arthroplasty. (**A**) Left femoral neck fracture. (**B**) Elevated C-reactive protein level and wound complications on postoperative day 14. (**C**) A change in the acetabular cup angle at 7 months postoperatively.

Detailed results are presented in Table 2. Vertical or horizontal migration of the acetabular cup and severe osteolysis around the acetabular cup were not observed during the follow-up period. In 1 (2.9%) case, a change in the acetabular cup angle was observed, which may be attributable to a surgical site infection (Fig. 1a–c). Progression of the radiolucent lines was not observed in all other cases. No signs of complications after BH were noted. During the evaluation of femoral stem stability, 21 out of 29 (72.4%) cases wherein surgery was performed using the cementless stem showed stable bone ingrowth, while stable fibrous fixation was noted in 8 (27.6%) cases. Unstable fixation or loosening was not observed in any case during the follow-up period; however, stable fixation was achieved in all 5 (100%) cases wherein surgery was performed using the cemented stem; loosening, migration, or subsidence of the stem was not detected postoperatively.

**Table 2:** Radiological outcomes.

	No. of cases
Acetabular cup	34
Total hip arthroplasty	23
Migration	0
Tilting	1
Osteolysis	0
Bipolar hemiarthroplasty	11
Proximal migration	0
Acetabular erosion	0
Femoral stem	
Dorr classification	37
A	3
B	25
C	9
Final follow-up	34
Stable (cementless)	29
Bone ingrowth	21
Fibrous fixation	8
Stable (cemented)	5
Unstable (migration, subsidence)	0

Dorr classification score evaluated using preoperative plain radiographs; other radiological outcomes were analyzed for all patients except for 3 patients who died within 1 year postoperatively.

### Clinical outcomes

The mean Harris hip scores were 46.7 (SD, ± 12.7) preoperatively (excluding those of patients with hip fractures) and 92.0 (SD, ± 6.2) at the final follow-up, with a statistically significant difference (*P* < 0.001). The mean final follow-up score (including those of patients with hip fractures) was 91.8 (excellent; Table 3).

**Table 3:** Clinical outcomes.

Clinical^#^			
	Preoperatively	Final follow-up	*P*
Harris hip score	46.7 ± 12.7	92.0 ± 6.2	< 0.001
Excellent (90–100)		31	
Good (80–89)		2	
Fair (70–79)		1	
Poor		0	

^#^Clinical outcomes were analyzed for all hips except the hips of 3 patients who died within 1 year postoperatively.

The average hospitalization duration was 25.4 days (SD, ± 10.5 days; range 10–63 days). The mean operative time was 103.7 min (SD, ± 28.2 min; range 90–225 min). The mean intraoperative blood loss volume was 310.3 mL (SD, ± 196.1 mL; range 100–1100 mL). Blood transfusion was required in 17 (45.9%) cases. None of the patients reported persistent inguinal or thigh pain postoperatively.

### Complications

Complications were observed within 1 year of surgery in 18 (52.9%) patients, 3 of whom had local complications (1 case of surgical site infection and 2 cases of postoperative dislocations). One of the patients with dislocations had schizophrenia and was conservatively treated, and there was no recurrence. Periprosthetic fractures or other local complications were also not noted.

Some patients had more than one complication. AKI-on-CKD was observed in 7 patients (38.9% of the 18 patients with complications within 1 year vs. 20.6% of the total 34 patients); urinary tract infections in 7 (38.9% vs. 20.6%); pneumonia in 2 (11.1% vs. 5.9%); atelectasis in 2 (11.1% vs. 5.9%); septic shock in 1 (5.6%; 2.9% of the total); cardiac arrest in 1 (5.6% vs. 2.9%); venous thromboembolism in 1 (5.6% vs. 2.9%); and pulmonary edema in 2 (5.6% vs. 5.9%). Three patients died of acute-on-CRF, septic shock, and cardiac arrest, respectively (Table 4).

**Table 4:** Incidence of early complications for patients undergoing hemodialysis after hip arthroplasty.

	Sex/age (years)	Early complications										
		SSI	Dislocation	Death	VTE	AKI	Septic shock	Cardiac arrest	Pneumonia	UTI	Other infections	Other complications
1	F/47									O		
2	F/51			O	O	O						
3	F/56					O						
4	M/57			O			O					
5	F/57										O (infectious colitis)	
6	F/57								O			O (atelectasis)
7	F/64									O		
8	M/64		O			O						
9	F/65									O		
10	M/68		O									O (atelectasis)
11	F/69					O				O	O (PMC)	
12	M/69					O						
13	M/71	O								O		
14	M/74			O				O				O (pulmonary edema)
15	F/74									O		
16	F/75					O						O (pulmonary edema)
17	F/79					O						
18	M/81								O	O		
Total		1	2	3	1	7	1	1	2	7	2	4

*O* complication occurred, *F* female, *M* male, *SSI* surgical site infection, *VTE* venous thromboembolism, *AKI* acute kidney insufficiency, *UTI* urinary tract infection, *PMC* pseudomembranous colitis.

Local complications were not observed at 1 year postoperatively. Nevertheless, 12 (35.3%) patients presented with general complications and some exhibited more than one complication, as follows: urinary tract infection was observed in 6 (50.0%; 19.4% of the total) patients; AKI-on-CKD in 4 (33.3%; 12.9% of the total); pneumonia in 3 (25.0%; 9.7% of the total); septic shock in 2 (16.7%; 6.5% of the total); cardiac arrest in 1 (8.3%; 3.2% of the total); acute cholecystitis in 1 (8.3%; 3.2% of the total); gastric ulcer bleeding in 1 (8.3%; 3.2% of the total); and pulmonary edema in 1 (8.3%; 3.2% of the total). Five patients died of AKI-on-CKD (3 patients), septic shock (1 patient), and cardiac arrest (1 patient) (Table 5).

**Table 5:** Incidence of late complications for patients undergoing hemodialysis after hip arthroplasty.

	Sex/age (years)	Late complications							
		Death	AKI	Septic shock	Cardiac arrest	Pneumonia	UTI	Other infections	Other complications
1	F/47						O		
2	F/56	O		O			O		
3	F/56								O (gastric ulcer bleeding)
4	F/57	O	O	O		O		O (ischemic colitis)	
5	F/57					O	O		
6	F/60	O			O				
7	F/64	O	O				O		O (acute cholecystitis)
8	F/65						O		
9	M/68	O	O						
10	M/68					O			
11	F/74						O		
12	F/75		O						O (pulmonary edema)
Total		5	4	2	1	3	6	1	3

*O* complication occurred, *F* female, *M* male, *AKI* acute kidney insufficiency, *UTI* urinary tract infection.

## Discussion

Femoral stem stability and satisfactory implantation of the acetabular cup were achieved in patients on dialysis who underwent hip arthroplasty. However, during the follow-up period, which lasted an average of 36.6 months (SD, ± 27.2 months), 8 patients died and 21 had significant complications. Moreover, the total dialysis duration was associated with the development of complications, indicating that general complications, including death, could occur after hip arthroplasty in CRF patients on dialysis, even when the orthopedic outcome of the surgery was outstanding.

Deposition of peri-implant beta 2-microglobulin amyloid in the pseudocapsule and pseudomembranous tissue in chronic hemodialysis patients results in the loosening of the prosthesis^26^. Furthermore, THA failure in patients on hemodialysis is attributable to the loosening of the prosthesis due to poor bone quality and high osteoclast activity, secondary to increased serum parathyroid hormone levels in CRF patients, thus leading to increased bone resorption and destabilization of bone implant fixation^27^. Nagoya et al. used an extensively porous-coated cementless stem for dialysis patients undergoing hip arthroplasty and reported a 100% fixation rate with bone ingrowth^10^. During this study, although many patients developed osteoporosis, the use of proximally porous-coated cementless stems or cemented stems enabled a firm fixation.

The main goal of hip arthroplasty is to restore normal ambulation and physical activity, which is known to be difficult in CRF patients^3,4^. In this study, clinical evaluations during the follow-up period revealed satisfactory improvements. In 1 (2.9%) case with a fair outcome, general weakness due to the worsening of CRF led to difficulty in ambulation at > 3 years postoperatively. However, the clinical scores represent the outcomes at the time of scoring, not the long-term progressive outcomes. Patients on dialysis often have comorbidities (e.g., bleeding tendencies, infections, vascular diseases) in addition to CKD; hence, worsening of these underlying conditions and other associated causes may also lead to death^7,28^. We observed 8 fatalities in total, with 3 patients dying within 1 year. Therefore, the mortality rate of patients who had undergone hip arthroplasty and are undergoing dialysis may be high and may increase with the worsening of underlying diseases and infections.

Generally, immunosuppression (from chronic dialysis), malnutrition, and the lack of erythropoietin increase the incidence of infection around the surgical and prosthesis sites^7–9,28,29^. Naito et al. reported surgical site infections in 12% of patients who underwent hip arthroplasty^8^. Lieberman et al. and Sakalkale et al. also reported postoperative infections in 19% and 13% of patients, respectively^5,7^. These rates are much higher than the 5-year post-THA infection rates in the general population, which range between 0.2 and 1.1%^30^. During this study, surgical site infection was observed in 1 patient at < 1 year postoperatively, which improved with antibiotic administration and local debridement. The same patient showed acetabular cup tilting, which was considered to have been caused by infection recurrence at 7 months postoperatively, suggesting that the superficial infection had progressed to a deep infection. In general, infection was observed in 12 of the 21 (57.1%) patients with complications, and patients on dialysis are more susceptible to infections, which can often be progressive. Although hip arthroplasty may lead to acceptable orthopedic outcomes for patients on dialysis, more caution is needed regarding the general complications. Hip arthroplasty may be considered an appropriate treatment alternative if performed after a comprehensive general preoperative evaluation.

This study had some limitations. It used a retrospective design and included follow-up data of a relatively small sample of patients on dialysis. Therefore, it is difficult to attribute a general clinical significance to the results of this study. Moreover, the results of the study groups were not compared with those of a control group. This limitation may be overcome by comparing results between patients who underwent THA and BA performed under the same conditions. Therefore, prospective and comparative studies with larger patient groups are required to validate our findings.

In conclusion, hip arthroplasty for chronic renal failure patients on dialysis showed excellent radiological and satisfactory clinical outcomes; however, it may be associated with postoperative complications. Thorough preoperative planning and an appropriate holistic approach to postoperative management are required to decrease the incidence of general complications in post-THA patients on dialysis.

## Data Availability

The datasets used and/or analyzed during the current study are available from the corresponding author on reasonable request.
